# Exergaming and cognitive functions in people with mild cognitive impairment and dementia: a meta-analysis

**DOI:** 10.1038/s41746-024-01142-4

**Published:** 2024-06-15

**Authors:** Joyce Y. C. Chan, Jiani Liu, Aaron T. C. Chan, Kelvin K. F. Tsoi

**Affiliations:** 1 JC School of Public Health and Primary Care, Faculty of Medicine, The Chinese University of Hong Kong, Hong Kong, China; 2 Stanley Ho Big Data Decision Analytics Research Centre, The Chinese University of Hong Kong, Hong Kong, China

**Keywords:** Dementia, Medical research

## Abstract

Exergaming is a combination of exercise and gaming. Evidence shows an association between exercise and cognition in older people. However, previous studies showed inconsistent results on the cognitive benefits of exergaming in people with cognitive impairment. Therefore, this study aims to examine the effect of exergaming intervention on cognitive functions in people with MCI or dementia. A systematic literature search was conducted via OVID databases. Randomized controlled trials (RCTs) examined the effect of an exergaming intervention on cognitive functions in people with MCI or dementia were included. Subgroup analyses were conducted according to the type of intervention and training duration. Twenty RCTs with 1152 participants were identified, including 14 trials for MCI and 6 trials for dementia. In people with MCI, 13 studies used virtual-reality (VR)-based exergaming. Those who received VR-based exergaming showed significantly better global cognitive function [SMD (95%CI) = 0.67 (0.23–1.11)], learning and memory [immediate recall test: 0.79 (0.31–1.27); delayed recall test: 0.75 (0.20–1.31)], working memory [5.83 (2.27–9.39)], verbal fluency [0.58 (0.12–1.03)], and faster in executive function than the controls. For people with dementia, all studies used video-based exergaming intervention. Participants with exergaming intervention showed significantly better global cognitive function than the controls [0.38 (0.10–0.67)]. Subgroup analyses showed that longer training duration generated larger effects. The findings suggest that exergaming impacts cognitive functions in people with MCI and dementia. Cognitive benefits are demonstrated for those with a longer training duration. With technological advancement, VR-based exergaming attracts the attention of people with MCI and performs well in improving cognitive functions.

## Introduction

The prevalence rate of mild cognitive impairment (MCI) and dementia in older persons is high worldwide^1–3^. The number of new cases is projected to continue to increase due to the ageing population^4^. MCI is defined as a preclinical and transitional stage between healthy ageing and dementia^5^. Around 5% to 13% of people with MCI converted to dementia annually^6^. Dementia is a condition that can be caused by different types of diseases in which the nerve cells of the brain are damaged over time and may lead to deterioration in cognitive functions^7^. Cognitive decline, including memory loss and deterioration of executive function, are the initial symptoms of MCI and dementia.

Exergaming, a combination of exercise and gaming, has become popular for training and rehabilitation in older people^8,9^. The players engage in physical and cognitive activities and play on a technology-based gaming system^8^. The exergames include exercise with games in video or virtual reality (VR) settings^10^. Studies either use existing commercial game systems, such as Wii and Kinect^11,12^, or self-developed virtual-reality (VR) games, such as VR-based cycling^13^. Exergaming provides cognitive stimulation and creates an interactive gamification environment that can stimulate multiple cognitive functions, including memory and executive function^11,14^. Such real-time gamification features motivate the participants to stay in the intervention^8,9^. Interactive video games with simple equipment nowadays are provided at an affordable price that can be easily set up in clinics or community centers. Exergaming in a safe environment is especially important for older people to conduct exercise and cognitive rehabilitation^8,9,15^.

Evidence shows that there is an association between physical exercise and certain domains of cognitive functions, such as memory and executive function, in older people^16–19^. However, other reviews did not find an effect of physical exercise on cognitive functions in people with cognitive impairment^20,21^. In addition, a study revealed that physical exercise with a longer duration had a larger effect on improving cognition than a shorter duration in people with dementia^19^. Compared with traditional physical exercises, exergaming interventions have been shown greater cognitive benefits, such as global cognitive function and executive function in people with cognitive impairment^22^. However, another study did not show such cognitive benefit with exergaming^23^. Some systematic reviews were performed, but they showed inconsistent results on the cognitive benefits of exergaming in people with cognitive impairment^8,24–27^. Also, they did not provide the details on types and training duration of exergaming intervention. Therefore, it is still unclear whether the effects of exergaming would be affected by the types and duration of an intervention.

Therefore, the objective of this systematic review and meta-analysis was to examine the effect of exergaming on cognitive functions, specifically the type and training duration in people with MCI and dementia.

## Results

### Literature search and study selection

**Fig. 1:**
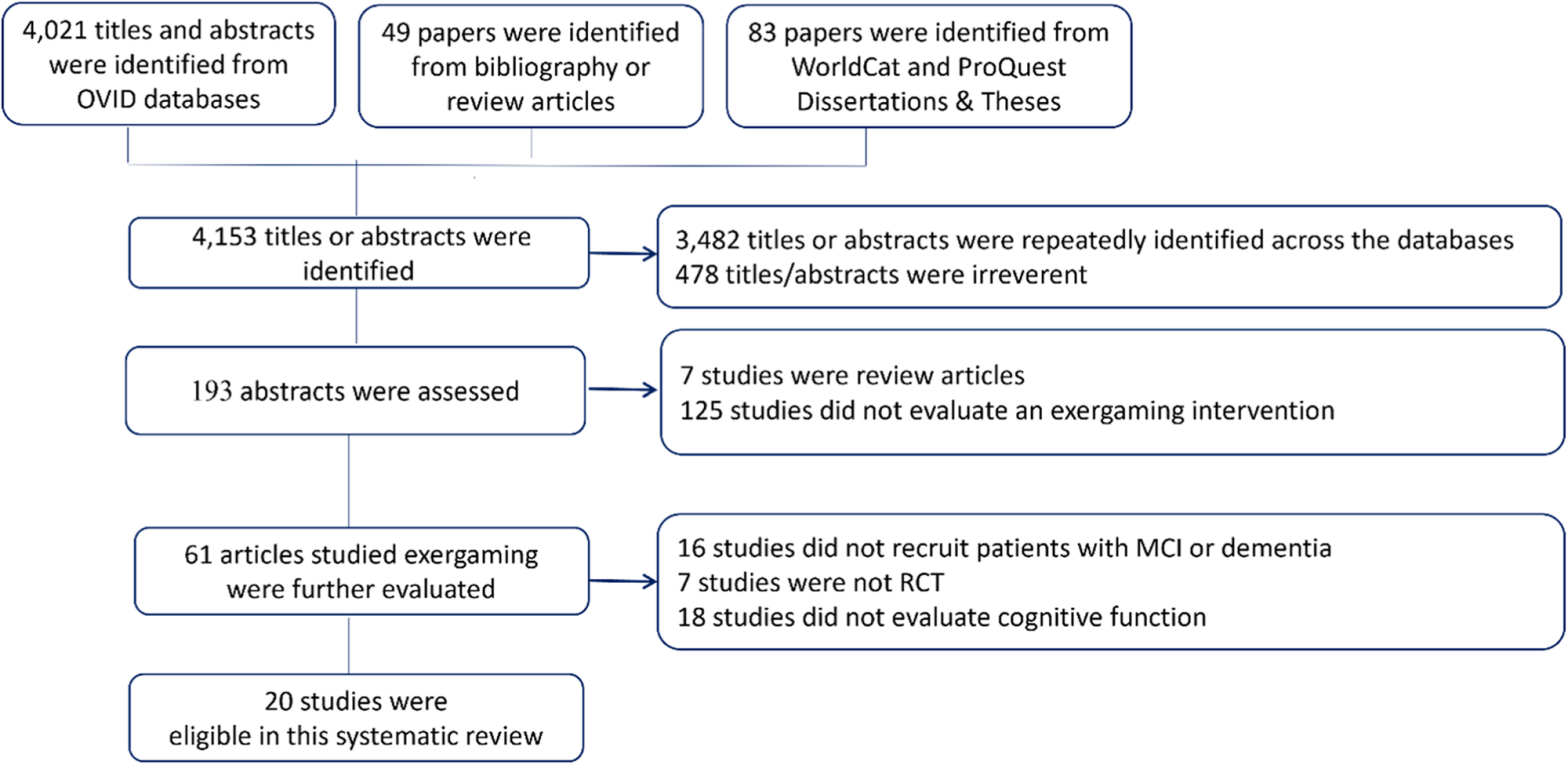
Flowchart of literature search.

A total of 4021 titles were identified from OVID databases, all titles were screened. An additional 49 articles were identified from review articles and bibliography, and 83 papers were identified from WorldCat and ProQuest Dissertations & Theses. After excluding the irrelevant titles, repeated titles across databases, review articles, and studies that did not evaluate an exergaming intervention, 61 articles related to exergaming intervention were further evaluated. Sixteen studies did not recruit participants with MCI or dementia; 7 studies were not RCT; and 18 studies did not evaluate cognitive function (Supplementary Table 2). As a result, 20 RCTs were eligible for this systematic review and meta-analysis^11–13,22,23,28–42^ (Fig. 1).

### Studies characteristics

Among these 20 RCTs, 14 of them recruited participants with MCI (*n* = 773), and six of them recruited participants with dementia (*n* = 379). The mean age of participants ranged from 67 to 87 years old, and the proportion of males ranged from 15% to 62% (Table 1). The year of publication of the included studies ranged from 2012 to 2023. Thirteen studies used VR-based exergaming intervention, and all of them were implemented in people with MCI. Seven studies used video-based exergaming intervention, and 6 of them were implemented in people with dementia. The list of exergaming interventions is shown in Table 2. Seventeen studies used a 2-arms study design, and three studies used a 3-arms study design. The length of intervention ranged from 4 weeks to 24 weeks, and the median length of intervention was 11 weeks. So, <12 weeks and ≥12 weeks were used as the cut-off in the subgroup analysis. Nine studies used a short training duration (<12 weeks), and 10 studies used a long training duration (≥12 weeks). The frequency of intervention ranged from 1 to 5 times per week, and the maximum length of each session ranged from 30 to 100 min. Eighteen studies recruited participants from community or day centres, one study recruited participants from an assisted living facility, and the remaining one study recruited participants from a hospital. Eight studies used the intention-to-treat principle, 10 studies used the pre-protocol principle, and the remaining two studies did not describe the method of result analysis. Four studies were assessed as high risk of bias in one of the ROB-2 domains, including deviations from intended interventions^41^, measurement of the outcome^33^, and selective reporting of results^23,28^ (Supplementary Table 3).

**Table 1:** Characteristics of included studies

Study ID (first author and year)	Country/Region	Mean age	Male %	No. of participants	Disease severity	Type of exergaming	Type of control	Training duration	No. of sessions
Amjad 2019^28^	Pakistan	–	–	44	MCI	VR-based	Exercise	6 weeks	30
Anderson-Hanley 2012^22^	USA	78.1	34	79	MCI	VR-based	Exercise	24 weeks	120
Choi 2019^29^	Korea	76.3	15	60	MCI	VR-based	Exercise	6 weeks	12
Delbroek 2017^30^	USA	86.9	20	20	MCI	VR-based	Usual Care	6 weeks	12
Hughes 2014^11^	USA	78.5	20	20	MCI	Video-based	Usual Care	24 weeks	12
Karssemeijer 2019^23^	Netherlands	80.0	54	76	Dementia	Video-based	Usual Care, Exercise	12 weeks	36
Liao 2019^31^	China	75.5	39	42	MCI	VR-based	Exercise	12 weeks	36
Liao 2020^32^	Taiwan	74.4	32	42	MCI	VR-based	Exercise	12 weeks	36
Liu 2022^12^	Taiwan	74.6	25	36	MCI	VR-based	Usual Care, Exercise	12 weeks	12
Mrakic-Sposta 2018^13^	Italy	73.3	40	12	MCI	VR-based	Usual Care	6 weeks	18
Palada 2012^33^	USA	80.0	27	22	Dementia	Video-based	Exercise	8 weeks	40
Palada 2017^34^	USA	73.0	19	30	Dementia	Video-based	Exercise	8 weeks	40
Park 2018^35^	Korea	67.0	54	78	MCI	VR-based	Cognitive Training	10 weeks	30
Schwenk 2016^36^	USA	77.8	42	78	MCI	VR-based	Usual Care	4 weeks	8
Tarnanas 2014^37^	Greece	70.5	38	73	MCI	VR-based	Usual Care, Cognitive Training	20 weeks	40
Thapa 2020^38^	Korea	72.6	18	68	MCI	VR-based	Usual Care	8 weeks	24
Torpil 2021^39^	Turkey	70.1	37	64	MCI	VR-based	Usual Care	12 weeks	24
van Santen 2020^40^	Netherlands	79.0	54	112	Dementia	Video-based	Usual Care	24 weeks	120
Wu 2023^41^	Korea	78.8	62	52	Dementia	Video-based	Exercise	12 weeks	36
Zheng 2022^42^	China	81.7	17	48	Dementia	Video-based	Usual Care	8 weeks	40

*MCI* mild cognitive impairment, *VR* virtual reality, *No.* number.

**Table 2:** List of exergaming interventions in the included studies

Type of exercise/game involved	Number of studies	Type of exergaming	Developers
Biking^13,22,23,40,41^	5	VR-based, Video-based	Tano and LongGood programs, Bike Labyrinth, Talesrunner IP & ExerHeart Self-developed
Tai Chi^12,32,33^	3	VR-based	Tano and LongGood programs, Microsoft Kinect
Walking and crossing obstacles^13,30,36^	3	VR-based	Self-developed
Resistance exercise^31,32^	2	VR-based	Tano and LongGood programs
Aerobic exercise^31–34^	2	VR-based, video-based	Tano and LongGood programs
Sport games^11,35^	2	VR-based, video-based	Nintendo Wii
Shooting^38,39^	2	VR-based	Microsoft Kinect, SY Innotech Inc., Busan
Boxing^39^	1	VR-based	Microsoft Kinect
Jet run^39^	1	VR-based	Microsoft Kinect
Skydiving^39^	1	VR-based	Microsoft Kinect
Traffic control^28^	1	VR-based	Microsoft Kinect
Mouse Mayhem^28^	1	VR-based	Microsoft Kinect
Kayak paddling exercise^29^	1	VR-based	Self-developed
Fruit Ninja^42^	1	Video-based	Microsoft Kinect
Dancing, movement^37^	1	VR-based	Self-developed
Running (Alchemist’s Treasure)^41^	1	Video-based	Talesrunner IP & ExerHeart

### Global cognitive function in people with MCI

**Fig. 2:**
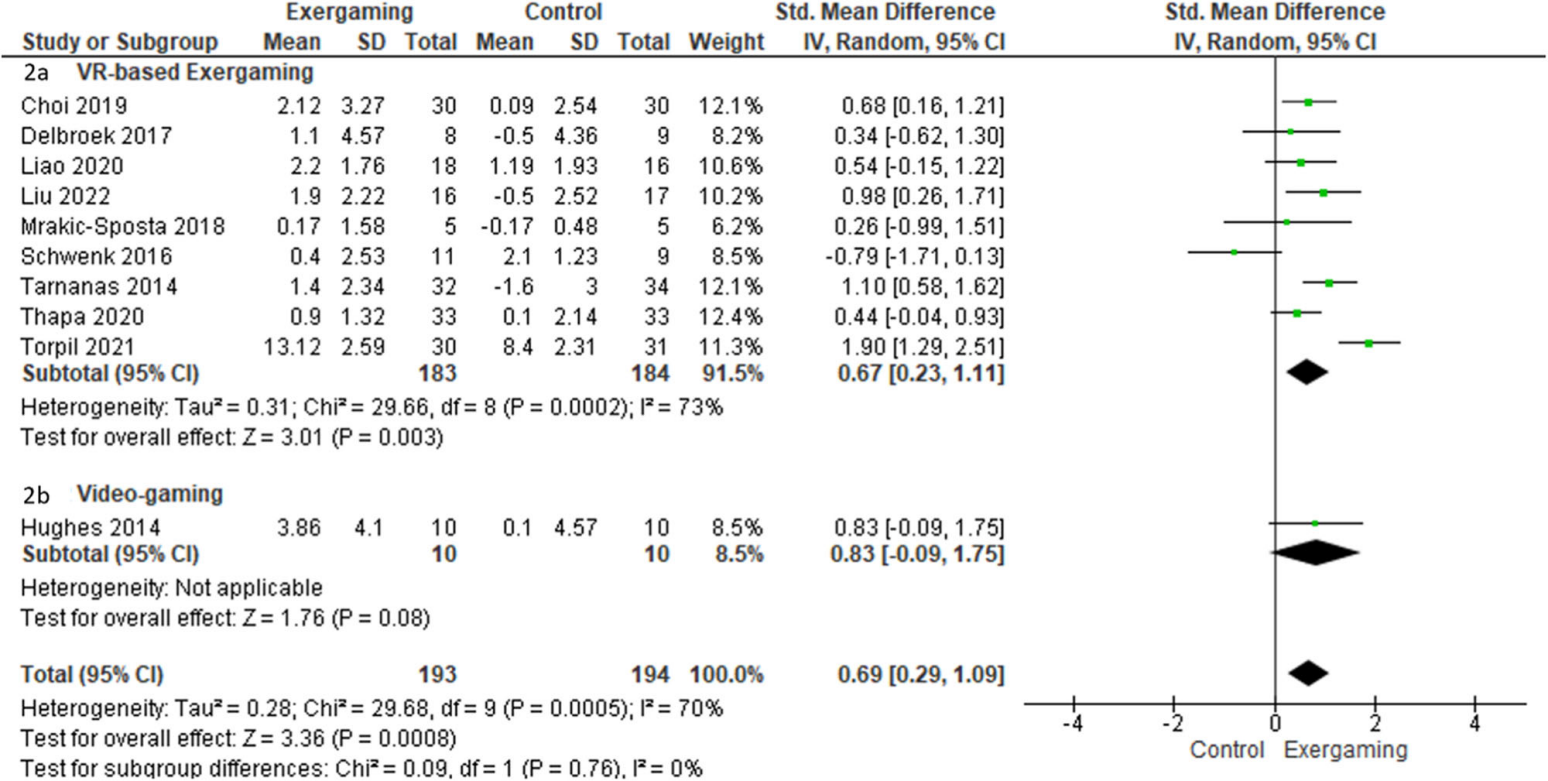
Effect of exergaming intervention on global cognitive function in people with mild cognitive impairment.

Ten cohorts evaluated the effects of exergaming intervention on global cognitive function in people with MCI. The heterogeneity was large (*I*^2^ = 70%), and a random-effects model was used (Fig. 2). The participants who received exergaming intervention showed significantly better global cognitive function than the controls (SMD = 0.69, 95% CI = 0.29–1.09, *k* = 10). Publication bias was assessed by funnel plot and egger test. The funnel plots did not show significant asymmetry (Supplementary Fig. 1). In subgroup analyses, 9 out of 10 cohorts used VR-based exergaming intervention. Participants with VR-based exergaming intervention showed a medium and significant effect on global cognitive function as compared with the controls (SMD = 0.67, 95% CI = 0.23–1.11, *k* = 9) (Table 3). Participants with a long training duration showed significantly better global cognitive function than the controls (SMD = 1.10, 95% CI = 0.53–1.57, *k* = 5). However, no significant difference was found between intervention and controls in participants with a short training duration (SMD = 0.28, 95% CI = −0.18 to 0.74, *k* = 5).

**Table 3:** Effects of exergaming intervention in people with mild cognitive impairment

Cognitive domains	Global cognitive function		Learning & memory				Working memory		Verbal fluency		Complex attention TMT-A (s)		Executive function TMT-B (s)	
			Immediate recall test		Delayed recall test									
Effects of exergaming	SMD (95% CI)	*k*	SMD (95% CI)	*k*	SMD (95% CI)	*k*	SMD (95% CI)	*k*	SMD (95% CI)	*k*	MD (95% CI)	*k*	MD (95% CI)	*k*
Main analysis	**0.69** **(0.29, 1.09)**	10	**0.79** **(0.31, 1.27)**	4	**0.75** **(0.20, 1.31)**	6	**5.83** **(2.27, 9.39)**	4	**0.58** **(0.12, 1.03)**	2	−0.65 (−2.05, 0.75)	5	**−9.71** **(−15.1, −4.29)**	**7**
Subgroup analysis														
(i) *Type of Exergaming*														
Video-based	0.83 (−0.09, 1.75)	1	**–**		**--**				**--**		**--**		**--**	
VR-based	**0.67** **(0.23, 1.11)**	9	**0.79** **(0.31, 1.27)**	4	**0.75** **(0.20, 1.13)**	6	**5.83** **(2.27, 9.39)**	4	**0.58** **(0.12, 1.03)**	**2**	−0.65 (−2.05, 0.75)	5	**−9.71** **(−15.1, −4.29)**	7
(ii) *Length of intervention*														
Short (<12 weeks)	0.28 (−0.18, 0.74)	5	0.56 (−0.72, 1.84)	1	0.83 (−0.34, 2.00)	2	**4.54** **(3.68, 5.40)**	1	0.20 (−1.04, 1.45)	1	−0.84 (−2.82, 1.13)	3	−4.30 (−9.71, 1.12)	4
Long (≥12 weeks)	**1.10** **(0.53, 1.57)**	5	**0.81** **(0.25, 1.37)**	3	**0.93** **(0.31, 1.54)**	4	**6.33** **(2.09, 10.6)**	3	**0.64** **(0.15, 1.13)**	1	−4.69 (−15.1, 5.73)	2	**−70.1** **(−131, −8.23)**	3
(iii) *Type of Control Group*														
Usual care	**0.52** **(0.07, 0.97)**	7	**0.51** **(0.06, 0.96)**	2	**1.31** **(0.65, 1.96)**	3	0.15 (−0.59, 0.89)	2	**0.58** **(0.12, 1.03)**	2	−0.92 (−2.84, 1.01)	4	−47.3 (−105, 10.9)	3
Active control	0.69 (0.09, 1.30)	5	**0.93** **(0.48, 1.37)**	3	0.14 (−0.17, 0.44)	5	**5.89** **(2.54, 9.23)**	4	0.25 (−0.22, 0.72)	1	−2.30 (−13.2, 8.56)	2	**−8.09** **(−14.1, −2.06)**	5

An increase in score indicated better cognitive functions in global cognitive function, learning and memory, working memory, and verbal fluency. A decrease in time score in TMT-A and TMT-B indicated better complex attention and executive function.

*VR* virtual reality, *MCI* mild cognitive impairment, *SMD* standardized mean difference, *MD* mean difference, *CI* confidence interval, *k* number of cohorts, *TMT-A* trail making test-*A*, *TMT-B* trail making test-B.

The effect sizes with statistical significance (at 95% confidence level) are highlighted in bold.

### Learning and memory in people with MCI

Four cohorts evaluated the effects of VR-based exergaming intervention with an immediate recall test. Participants who received VR-based exergaming intervention showed significantly higher scores in the immediate recall test than the controls (SMD = 0.79, 95% CI = 0.31–1.27, *k* = 4) (Supplementary Fig. 2a). In subgroup analyses, participants with a long training duration showed significantly higher scores in immediate recall test than the controls (SMD = 0.81, 95% CI = 0.25–1.37, *k* = 3). Participants with exergaming intervention showed significantly higher scores in the immediate recall test than the usual care (SMD = 0.51, 95% CI = 0.06–0.96, *k* = 2) and active controls (SMD = 0.93, 95% CI = 0.48–1.37, *k* = 3).

Six cohorts evaluated the effects of VR-based exergaming intervention with a delayed recall test. The participants who received VR-based exergaming intervention showed significantly higher scores in the delayed recall test than the controls (SMD = 0.75, 95% CI = 0.20–1.31, *k* = 6) (Supplementary Fig. 2b). In subgroup analyses, participants with a long training duration showed significantly higher scores in delayed recall test than the controls (SMD = 0.93, 95% CI = 0.31–1.54, *k* = 4). However, no significant difference was found in participants with a short training duration (SMD = 0.83, 95% CI = −0.34 to 2.00, *k* = 2).

### Working memory in people with MCI

Four cohorts evaluated the effects of VR-based exergaming intervention on working memory. Participants with VR-based exergaming intervention showed significantly better working memory than the controls (SMD = 5.83, 95% CI = 2.27–9.39, *k* = 4) (Supplementary Fig. 3). In subgroup analyses, participants with either long or short training duration showed significantly better working memory than the controls. The participants with VR-based exergaming intervention showed significantly better performance in working memory than the active controls (SMD = 5.89, 95% CI = 2.54–9.23, *k* = 4).

### Verbal fluency in people with MCI

Two cohorts evaluated the effects of VR-based exergaming intervention on verbal fluency. Participants who received VR-based exergaming intervention showed significantly higher scores on the verbal fluency test than the controls (SMD = 0.58, 95% CI = 0.12–1.03, *k* = 2) (Supplementary Fig. 4).

### Complex attention in people with MCI

Five cohorts evaluated the effects of VR-based exergaming intervention on complex attention with TMT-A. No significant difference was found between the intervention and controls (MD = −0.65, 95% CI = −2.05 to 0.75, *k* = 5) (Supplementary Fig. 5).

### Executive function in people with MCI

Seven cohorts evaluated the effects of VR-based exergaming intervention on executive function with TMT-B. Participants who received VR-based exergaming intervention showed significantly decreased time in TMT-B than the controls (MD = −9.71, 95% CI = −15.1 to −4.29, *k* = 7) (Supplementary Fig. 6). In subgroup analyses, participants with a long training duration showed significantly better executive function than the controls (MD = −70.1, 95% CI = −131 to −8.23, *k* = 3). However, no significant difference was found in participants with a short training duration. VR-based exergaming intervention group showed a significant decrease in time in TMT-B as compared with the active controls (MD = −8.09, 95% CI = −14.1 to −2.06, *k* = 5).

### Global cognitive function in people with dementia

**Fig. 3:**
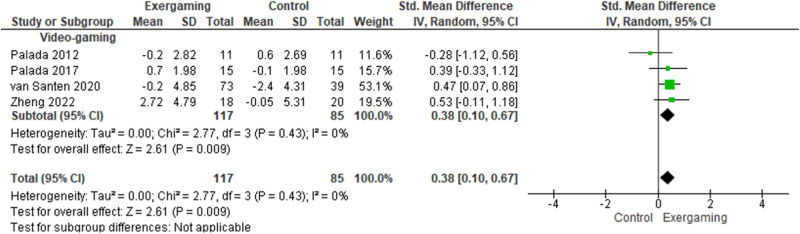
Effect of exergaming intervention on global cognitive function in people with dementia.

Four cohorts evaluated the effects of video-based exergaming intervention on global cognitive function in people with dementia. Participants who received exergaming intervention showed a small and significant effect on global cognitive function as compared with the controls (SMD = 0.38, 95% CI = 0.10–0.67, *k* = 4) (Table 4, Fig. 3). In subgroup analyses, participants with a long training duration showed significantly better global cognitive function than the controls (SMD = 0.47, 95% CI = 0.17–0.86, *k* = 1). However, no significant difference between intervention and controls was found in participants with a short training duration (SMD = 0.29, 95% CI = −0.17 to 0.74, *k* = 3).

**Table 4:** Effects of exergaming intervention in people with dementia

Cognitive domains	Global cognitive function		Learning & memory				Working memory		Verbal fluency		Complex attention TMT-A (s)		Executive function TMT-B (s)	
			Immediate recall test		Delayed recall *t*									
Effects of exergaming	SMD (95% CI)	*k*	SMD (95% CI)	*k*	SMD (95% CI)	*k*	SMD (95% CI)	*k*	SMD (95% CI)	*k*	MD (95% CI)	*k*	MD (95% CI)	*k*
Main analysis	**0.38** **(0.10, 0.67)**	**4**	–		–		–		–		−2.50 (−25.8, 20.8)	1	16.2 (−6.29, 38.7)	1
*Subgroup analyses*														
(i) Type of exergaming														
Video-based	**–**										−2.50 (−25.8, 20.8)	1	16.2 (−6.29, 38.7)	1
VR-based	**0.38** **(0.10, 0.67)**	4											**–**	
(ii) Length of intervention														
Short (<12 weeks)	0.29 (−0.17, 0.74)	3											**–**	
Long (≥12 weeks)	**0.47** **(0.17, 0.86)**	1									−2.50 (−25.8, 20.8)	1	16.2 (−6.29, 38.7)	1
(iii) Type of control group													–	
Usual care	**0.49** **(0.15, 0.82)**	2									−2.50 (−25.8, 20.8)	1	16.2 (−6.29, 38.7)	1
Active control	−0.09 (−0.56, 0.75)	2											**--**	

An increase in score indicated better cognitive functions in global cognitive function. A decrease in time score in TMT-A and TMT-B indicated better complex attention and executive function.

*VR* virtual reality, *MCI* mild cognitive impairment, *SMD* standardized mean difference, MD mean difference, *CI* confidence interval, *k* number of cohorts, *TMT-A* trail making test-B, *TMT-B* trail making test-B.

The effect sizes with statistical significance (at 95% confidence level) are highlighted in bold.

### Complex attention and executive function in people with dementia

One cohort evaluated the effects of VR-based exergaming intervention on complex attention and executive function in people with dementia. No significant difference was found between intervention and controls in TMT-A (MD = −2.50 (95% CI = −25.8 to 20.8) and TMT-B (MD = 16.2 (95% CI = −6.29 to 38.7). No study evaluated the effect of learning and memory, working memory, and verbal fluency in people with dementia.

### Sensitivity analyses

Sensitivity analyses were conducted according to individual cognitive tests. In global cognitive function in people with MCI, the results of MMSE and Loewenstein Occupational Therapy Cognitive Assessment-Geriatric showed a significant positive effect of exergaming intervention (Supplementary Table 4). Another sensitivity analysis was conducted to exclude a study with a high risk of bias^33^, and a significant positive effect remained on global cognitive function in people with dementia (SMD = 0.47, 95% CI = 0.16–0.77, *k* = 3).

## Discussion

Exergaming impacts cognitive functions in people with MCI and dementia in terms of global cognitive function, learning and memory, working memory, verbal fluency, and executive function. Cognitive benefits were demonstrated for those with at least 12 weeks training duration. Video exergaming shows cognitive benefits for people with dementia. With technological advancement, VR-based exergaming attracts the attention of people with MCI and performs well in improving cognitive functions.

The VR-based intervention provides a multisensory, immersive environment and a sensation inside the virtual environment for the users^43^. Most of the included studies adopted the existing VR-based exergaming programs^12,28,31,32,35,38,39^, and some of the VR programs were designed by the researchers^13,36,37^. Two studies measured the change in brain activity by electroencephalography in MCI patients. They found that there was a decrease in activation of the prefrontal areas and the partial and temporal regions after exergaming intervention^32,38^. The effects of VR-based exergaming may be due to the training that can improve brain activation and enhance functional brain plasticity^31,32,37,38^. However, even though exergaming shows benefits in various cognitive domains in people with MCI, no effect was found in complex attention. Therefore, further understanding of the exact mechanism of how the training improves or affects brain functions is suggested.

The use of VR-based exergaming is less common in people with dementia. The deterioration in cognitive functions, including memory, attention span, and executive function, may limit the ability of people with dementia to interact with the VR-environment. A proof-of-concept study investigated the use of VR-based exergaming for people with dementia and suggested that various customized designs of VR-based exergaming were required to accommodate the needs of people with dementia. For example, minimize the complexity of making actions, set the time of each activity to be less than 5 min to avoid fatigue, use high contrast tasks, and provide verbal instructions during the process^44^. Eisapour et al. 2020 found that VR-based exergaming with customized design was feasible in people with dementia. However, Eisapour et al. 2020 only recruited six participants with dementia and did not evaluate the effect of VR-based exergaming on cognition or functioning^44^. Therefore, further evaluation of VR-based exergaming in a larger sample of people with dementia is suggested.

A review article suggested that there was inadequate evidence for longer intervention could lead to greater improvements in function^25^. However, Zhao et al. (2020) did not perform any analysis on intervention duration^25^. In this study, the duration of exergaming intervention of at least 12 weeks was shown to be a better strategy to improve cognitive functions than those with a shorter period of exergaming. Besides, an expert panel rated the most adapted frequency of exergaming intervention in people with MCI and dementia as two to four times a week^45^. The frequency of intervention in 60% of the included studies was in-between 2 and 3 times per week. Thus, an exergaming intervention that lasts 2–3 times per week and lasts for at least 12 weeks may be useful and feasible in people with MCI and dementia.

Previous studies demonstrated the benefits of physical exercise and cognitive training in people with cognitive impairment^17,18,46,47^. In this study, the participants who received exergaming intervention showed benefits in learning and memory, working memory, and executive function than the active controls, such as traditional physical exercise and cognitive training. Exergaming requires players to carry out multiple tasks at a time and interact with computer games. The games facilitate different directions of comprehensive cognitive training, including working memory and executive function^32,33^. The strategies in exergaming may train cognitive abilities and mental flexibility that is stronger than traditional training.

Literature suggests that cognitive functions need to be continuously stimulated^48^. Exergaming creates pleasure and enjoyment; an interesting and attractive platform can help to maintain the motivation of older people to continue the training^30,37^. Besides, there were very few adverse events reported in the included studies. So, exergaming provides a safe environment to conduct training and rehabilitation for older people with cognitive impairment. A study reported that all the participants with MCI felt a better performance in their real life after VR-based exergaming intervention^13^. The elicited self-perceived improvement can help to motivate the participants to continue the exergaming intervention.

Some studies suggest that exergaming interventions are feasible to perform at home^30,34–36^. An included study evaluated an 8-week home-based caregiver-supervised Wii-Fit exercise program for people with dementia, but nil improvement in cognitive function was shown^34^. This may be due to the intervention duration being short. Further research on the protocol and effect of home-based exergaming intervention is suggested.

This study combined updated evidence to evaluate the effects of exergaming intervention across different cognitive domains in people with MCI and dementia, but there are some limitations. First, there is no standard protocol for conducting the intervention with exergaming. The included studies may have different designs on exergaming. Heterogeneity across the intervention is uncontrollable. Therefore, a random-effects model has been applied to handle the potential risk of heterogeneity. Second, studies were interpreted in different subgroup analyses, such as type and duration of exergaming. These subgroup analyses demonstrated the potential directions of further development of a standard protocol, but, unfortunately, the analyses were limited by a relatively small sample size. Third, studies used different types of outcome measurements, although we conducted sensitivity analyses on individual tests, substantial heterogeneity across the included studies was not fully interpreted. Fourth, studies recruited participants from different types of clinical and community-based settings, and used different training durations and frequencies, so clinical heterogeneity exists. Fifth, the data analysis was based on published studies, some unpublished studies may not have been identified through literature search, so publication bias was inevitable.

Exergaming impacts cognitive functions in people with MCI and dementia. Cognitive benefits are demonstrated for those with a longer training duration. With the advancement of technology, VR-based exergaming may further engage a higher participation rate. Further research on the effect of VR-based exergaming in people with dementia is recommended.

## Methods

This systematic review and meta-analysis followed the standard guidelines of the Cochrane Handbook^49^, and the reporting items followed Preferred Reporting Items for Systematic Reviews and Meta-Analyses (PRISMA)^50^. This meta-analysis was registered in the PROSPERO as CRD42022378761. During the process of data extraction, we further modified the outcomes with more domains of cognition, including global cognitive function, learning and memory, working memory, verbal fluency, complex attention, and executive function.

### Search strategy

Comprehensive literature searches were performed in the OVID databases, including MEDLINE, EMBASE, APA PsycINFO, PubMed, and CHIAHL, from the earliest available dates stated in each database until March 31, 2023. Searching keywords including “exergaming”, “exercise”, “virtual-reality”, “virtual-reality intervention”, “VR-based intervention”, “VR exercise”, “VR training”, “Wii”, “cyber”, “gaming”, “video game”, “Kinect”, “Xbox”, “dance”, and “bicycle” were searched with “dementia”, “cognitive impairment”, “mild cognitive impairment”, “MCI”, and “Alzheimer”, and also “trial”, “study”, and “random” (Supplementary Table 1). Bibliographies of review articles and studies were screened. Grey literature and dissertations were searched through WorldCat and ProQuest Dissertations & Theses A&I. Randomized controlled trials (RCTs) that evaluated the effect of an exergaming intervention on cognitive functions in people with MCI or dementia were included.

### Inclusion and exclusion criteria

RCTs were included if they (i) recruited participants with MCI or dementia in any type of clinical or community setting; (ii) evaluated any type of exergaming intervention, such as video gaming or VR-based exergaming and compared with a non-exergaming intervention as control; (iii) measured the change of cognitive score between pre-and-post intervention; and (iv) reported at least one of the cognitive domains, including global cognitive function, learning and memory, working memory, verbal fluency, complex attention, and executive function. Studies were excluded if they focused on cognitive decline in patients with Parkinson’s disease, stroke, Huntington’s disease, epilepsy, multiple sclerosis, diabetes, or psychiatric illness.

### Data extraction

Two investigators (J.L. and A.T.C.) performed the literature search and data extraction independently. A data extraction form was designed to store the data from the original literature. Data collected included the year of publication, study location, mean age, the proportion of males, number of participants, name of intervention, type of controls, duration of intervention, name, and score of the cognitive tests, method of data analysis, and number of adverse events. When discrepancies were found regarding study eligibility or data extraction, a third investigator (J.Y.C.) would make the final decision.

### Interventions and outcomes

Exergaming interventions were classified into two major categories, including (i) VR-based, and (ii) video-based. Studies were also grouped into different control arms, including (i) usual care; and (ii) active controls. Usual care included a waitlisted or education group, and the active controls included physical exercise, cognitive training, and combined exercise and cognitive training. The primary outcomes of this study were the change of pre-and-post intervention scores from cognitive tests that measure global cognitive function, learning and memory, working memory, verbal fluency, complex attention, and executive function.

Global cognitive function was measured by any type of multi-domain cognitive tests such as the Mini-mental State Examination (MMSE)^51^ and Montreal Cognitive Assessment^52^. Learning and memory were measured by any type of verbal memory test, such as the California Verbal Learning Test^53^. Working memory was assessed by any type of working memory test such as digit span^54^. Verbal fluency was assessed by any type of verbal fluency test, such as sematic fluency^55^. Higher scores of global cognitive function, learning and memory, working memory, and verbal fluency indicate better cognitive functions. If a study reported two or more assessment tools for the same cognitive domain, we would include the most frequently used assessment tools in the meta-analysis. Complex attention was measured by Trail Making Test-A (TMT-A), and executive function was measured by Trail Making Test-B (TMT-B)^56^. The performance of TMT-A and TMT-B was measured by time spent on the test (seconds). A faster time score in TMT-A and TMT-B indicates better functioning.

### Risk of bias

Potential risks of bias in each included study were assessed by the revised Cochrane risk-of-bias tool for randomized trials (RoB-2)^57^. The RoB-2 measures 6 items of risk-of-bias, including (i) bias arising from the randomization process; (ii) bias due to deviations from intended interventions; (iii) bias due to missing outcome data; (iv) bias in measurement of the outcome; (v) bias in selection of the reported result; and (vi) overall bias.

### Data synthesis and statistical analysis

Standardized mean differences (SMDs) with confidence interval (95% CI) were applied to calculate the combined results of different cognitive tests. Using conventional definitions, SMD estimates of 0.20, 0.50, and 0.80 were considered as small, medium, and large, respectively^58^. The trials using the same cognitive tests were combined by mean difference (MD). A random-effects model was applied, and statistical heterogeneity was assessed by *I*^2^ ^59,60^. A forest plot was used to graphically present the results of the meta-analysis. Meta-analyses were performed to combine the effect sizes in Review Manager (Version 5.4)^61^. Publication bias was assessed by funnel plots and Egger’s Test by using the dmetar package in R version 4.2.2^62–65^.

### Subgroup and sensitivity analyses

Subgroup analyses in each cognitive domain were performed according to (i) type of exergaming intervention, i.e. VR-based and video-based; (ii) length of intervention, i.e. short and long, we would use the median length of intervention of the included studies as the cut-off; and (iii) type of controls, i.e. usual care and active controls. Sensitivity analyses were conducted according to (i) risk of bias of the included studies; and (ii) individual cognitive tests.

### Supplementary information

Supplementary table 1,2,3,4, Supplementary Figure 1,2,3,4,5,6

## Data Availability

Data collected and used in this meta-analysis can be requested from the corresponding author.
