# Supplementary material for: Exergaming and cognitive functions in people with mild cognitive impairment and dementia: a meta-analysis
Source: NPJ Digit Med. 2024 Jun 15;7:154. doi: 10.1038/s41746-024-01142-4 (PMC11180097; doi:10.1038/s41746-024-01142-4)

Supplementary Table 1. Search Strategies

Medline search via Ebscohost<br>1. AB (dementia OR cognitive impair* OR mild cognitive impair OR MCI OR Alzheimer*)<br>2. AB (exergaming OR exercise OR Wii OR cyber* OR gaming OR virtual-reality OR virtual-reality intervention OR VR-based intervention OR VR exercise OR VR train* OR Kinect OR Xbox OR video game OR bicycle OR dance<br>3. AB (trial OR study OR random*)<br>4. 1+2+3
Embase<br>1. (dementia or cognitive impair* or mild cognitive impair or MCI or Alzheimer*).ab.<br>2. (exergaming or exercise or Wii or cyber* or gaming or virtual-reality or virtual-reality intervention or VR-based intervention or VR exercise or VR train* or Kinect or Xbox or video game or bicycle or dance).ab.<br>3. (trial or study or random*).ab.<br>4. 1+2+3
APA PsycINFO<br>1. (dementia or cognitive impair* or mild cognitive impair or MCI or Alzheimer*).ab.<br>2. (exergaming or exercise or Wii or cyber* or gaming or virtual-reality or virtual-reality intervention or VR-based intervention or VR exercise or VR train* or dance or Kinect or Xbox or video game or bicycle).ab.<br>3. (trial or study or random*).ab.<br>4. 1+2+3
CINAHL<br>1. AB (dementia OR cognitive impair* OR mild cognitive impair OR MCI OR Alzheimer*)<br>2. AB (exergaming OR exercise OR Wii OR cyber* OR gaming OR virtual-reality OR virtual-reality intervention OR VR-based intervention OR VR exercise OR VR train* OR Kinect OR Xbox OR video game OR bicycle OR dance)<br>3. AB (trial OR study OR random*)<br>4. 1+2+3
Pubmed<br>1. AB/Title (dementia OR cognitive impair* OR mild cognitive impair OR MCI OR Alzheimer*)<br>2. AB/Title (exergaming OR exercise OR Wii OR cyber* OR gaming OR virtual-reality OR virtual-reality intervention OR VR-based intervention OR VR exercise OR VR train* OR Kinect OR Xbox OR video game OR bicycle OR dance)<br>3. AB/Title (trial OR study OR random*)<br>4. 1+2+3

Supplementary Table 2. List of Excluded Studies

First Author & Year	Title	Reason for Exclusion
Anderson-Hanley_2018	The interactive Physical and Cognitive Exercise System (iPACES™): effects of a 3-month in-home pilot clinical trial for mild cognitive impairment and caregivers	Non-RCT
Bamidis_2015	Gains in cognition through combined cognitive and physical training: the role of training dosage and severity of neurocognitive disorder	Non-RCT
Barnes_2013	The Mental Activity and eXercise (MAX) Trial. A Randomized Controlled Trial to Enhance Cognitive Function in Older Adults	Did not recruit participants with MCI or dementia
Ben-Sadoun 2016	Physical and Cognitive Stimulation Using an Exergame in Subjects with Normal Aging, Mild and Moderate Cognitive Impairment.	Non-RCT
Brummel 2014	Feasibility and safety of early combined cognitive and physical therapy for critically ill medical and surgical patients: the Activity and Cognitive Therapy in ICU (ACT-ICU) trial	Did not recruit participants with MCI or dementia
Cano-Manas 2020	Effects of video-game based therapy on balance, postural control, functionality, and quality of life of patients with subacute stroke: A randomized controlled trial.	Nil cognitive outcome
Chao 2015	Physical and psychosocial effects of Wii fit exergames use in assisted living residents: A pilot study.	Nil cognitive outcome
Chiang 2012	Using Xbox 360 kinect games on enhancing visual performance skills on institutionalized older adults with wheelchairs	Did not recruit participants with MCI or dementia
Cho 2014	The effects of virtual reality-based balance training on balance of the elderly.	Did not recruit participants with MCI or dementia
Cicek 2020	Interactive video game-based approaches improve mobility and mood in older adults: a nonrandomized, controlled trial.	Did not recruit participants with MCI or dementia
Duque 2013	Effects of balance training using a virtual-reality system in older fallers.	Nil cognitive outcome

Eisapour 2020	Participatory design and evaluation of virtual reality games to romote engagement in physical activity for people living with dementia.	Nil cognitive outcome
Ellmers 2018	Recalibrating disparities in perceived and actual balance abilities in older adults: a mixed-methods evaluation of a novel exergaming intervention.	Non-RCT
Ferraz 2018	The effects of functional training, bicycle exercise, and exergaming on walking capacity of elderly patients with Parkinson disease: A pilot randomized controlled single-blinded trial.	Nil cognitive outcome
Gregory_2016	Physical and Cognitive Stimulation Using an Exergame in Subjects with Normal Aging, Mild and Moderate Cognitive Impairment	Nil cognitive outcome
Hsieh_201	The Effectiveness of a Virtual Reality-Based Tai Chi Exercise on Cognitive and Physical Function in Older Adults with Cognitive Impairment	Non-RCT
Jahouh_2021	Impact of an Intervention with Wii Video Games on the Autonomy of Activities of Daily Living and Psychological–Cognitive Components in the Institutionalized Elderly	Did not recruit participants with MCI or dementia
Janssen 2013	A Preliminary Study on the effectiveness of exergame Nin tendo wii Fit Plus on the balance of nursing home residents.	Non-RCT
Jorgensen 2013	Efficacy of Nintendo Wii training on mechanical leg muscle function and postural balance in community-dwelling older adults: a randomized controlled trial.	Nil cognitive outcome
Jung 2015	Kinematic effect of Nintendo Wii(TM) sports program exercise on obstacle gait in elderly women with falling risk.	Nil cognitive outcome
Keogh 2014	Physical and psychosocial function in residential aged-care elders: effect of Nintendo Wii sports games.	Non-RCT
Liao 2021	Effect of exergaming versus combined exercise on cognitive function and brain activation in frail older adults: A randomised controlled trial	Did not recruit participants with MCI or dementia
Maillot 2014	The braking force in walking: agerelated differences and improvement in older adults with exergame training.	Did not recruit participants with MCI or dementia
Monteiro-Junio 2017	Acute effects of exergames on cognitive function of institutionalized older persons: a single-blinded, randomized and controlled pilot study.	Did not recruit participants with MCI or dementia
Montero-Alía 2019	Controlled trial of balance training using a video game console in community-dwelling older adults.	Nil cognitive outcome

Mugueta-Aguinaga 2017	FRED: exergame to prevent dependence and functional deterioration associated with ageing. A pilot three-week randomized controlled clinical trial.	Did not recruit participants with MCI or dementia
Ogawa 2020	Effects of exergaming on cognition and gait in older adults at risk for falling.	Did not recruit participants with MCI or dementia
Pichierri 2012	A cognitive-motor intervention using a dance video game to enhance foot placement accuracy and gait under dual task conditions in older adults: a randomized controlled trial.	Nil cognitive outcome
Portela 2011	Wiitherapy on seniors: effects on physical and metal domains	Did not recruit participants with MCI or dementia
Rendon 2012	The effect of virtual reality gaming on dynamic balance in older adults.	Nil cognitive outcome
Rica 2020	Effects of a Kinect-based physical training program on body composition, functional fitness and depression in institutionalized older adults.	Nil cognitive outcome
Rogan 2016	Sensory-motor training targeting motor dysfunction and muscle weakness in long-term care elderly combined with motivational strategies: a single blind randomized controlled study.	Nil cognitive outcome
Sato 2015	Improving walking, muscle strength, and balance in the elderly with an exergame using Kinect: a randomized controlled trial.	Did not recruit participants with MCI or dementia
Song 2015	Effect of virtual reality games on stroke patients' balance, gait, depression, and interpersonal relationships.	Nil cognitive outcome
Taylor 2018	Exergames to improve the mobility of long-term care residents: a cluster randomized controlled trial.	Nil cognitive outcome
Tollar 2019	Vastly different exercise programs similarly improve parkinsonian symptoms: A randomized clinical trial.	Nil cognitive outcome
Tsuda 2016	A feasibility study of virtual reality exercise in elderly patients with hematologic malignancies receiving chemotherapy.	Did not recruit participants with MCI or dementia
Valiani 2017	A new adaptive homebased exercise technology among older adults living in nursing home: a pilot study on feasibility, acceptability and physical performance.	Non-RCT

Vieira-Gomes 2018	Feasibility, safety, acceptability, and functional outcomes of playing Nintendo Wii Fit PlusTM for frail older adults: a randomized feasibility clinical trial.	Did not recruit participants with MCI or dementia
Wiloth 2018	Motor-cognitive effects of a computerized game-based training method in people with dementia: a randomized controlled trial.	Nil cognitive outcome
Yeşilyaprak 2016	Comparison of the effects of virtual reality-based balance exercises and conventional exercises on balance and fall risk in older adults living in nursing homes in Turkey.	Nil cognitive outcome
Yoon 2015	Effect of virtual reality-based rehabilitation on upper-extremity function in patients with brain tumor: controlled trial.	Did not recruit participants with MCI or dementia

Abbreviations: MCI, Mild cognitive impairment; RCT, randomized controlled trial

Supplementary Table 3. Risk of Bias of Included Studies

	Bias arising from the randomization process	Bias due to deviations from intended interventions	Bias due to missing outcome data	Bias in measurement of the outcome	Bias in selection of the reported result	Overall Bias
Amjad 2019	L	S	S	L	H	H
Anderson-Hanley 2012	S	L	L	L	L	S
Choi 2019	L	L	L	L	L	L
Delbroek 2017	L	L	L	L	L	L
Hughes 2014	L	L	L	L	L	L
Karssemeijer 2019	L	S	L	S	H	H
Liao 2019	L	L	S	L	L	S
Liao 2020	L	L	S	L	L	S
Liu 2022	L	L	S	L	L	S
Mrakic-Sposta 2018	L	S	S	L	L	S
Palada 2012	L	S	L	H	L	H
Palada 2017	L	L	L	L	L	L
Park 2018	L	S	L	L	L	S
Schwenk 2016	L	S	L	L	L	S
Tarnanas 2014	L	L	L	L	L	L
Thapa 2020	L	L	L	L	L	L
Torpil 2021	L	S	L	L	L	S
van Santen 2020	S	S	L	L	L	S
Wu 2023	L	H	S	L	L	H
Zheng 2022	L	S	L	L	L	S

L, Low risk; S, Some Concerns: H, High risk

Supplementary Table 4. Mean Difference between Exergaming and Control Group on Different Cognitive Tests

Cognitive Domains	Name of Cognitive Test	Number of Cohorts	MD (95% CI)
Participants with MCI			
Global Cognitive Function	Montreal Cognitive Assessment	5	1.02 (-0.47, 1.51)
	Mini-Mental State Examination	4	**1.59 (0.19, 2.88)**
	Computerized Assessment of Mild Cognitive Impairment	1	3.76 (-0.05, 7.57)
	Loewenstein Occupational Therapy Cognitive Assessment-Geriatric	1	**4.72 (3.49, 5.95)**
Immediate Recall Test	Rey Auditory Verbal Learning Test	3	**0.89 (0.56, 1.22)**
	Chinese Version of the California Verbal Learning Test	1	0.56 (-0.13, 1.25)
Delayed Recall Test	Rey Auditory Verbal Learning Test	4	0.79 (-0.03, 1.61)
	Chinese Version of the California Verbal Learning Test	2	**0.70 (0.21, 1.20)**
Working Memory	Digit Span backward	3	0.46 (-2.28, 1.20)
	n-back (one-back)	1	0.57 (-0.13, 1.27)
Participants with Dementia			
Global Cognitive Function	Mini-Mental State Examination	4	**1.11 (0.16, 2.06)**

Abbreviations: MD, Mean Difference; CI, Confidence Interval

Supplementary Figure 1. Funnel Plot of Publication Bias

a. Global Cognitive Function (MCI)

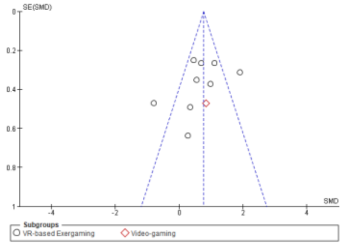

Egger's Intercept = -2.10, p = .34

b. Immediate Recall Test (MCI)

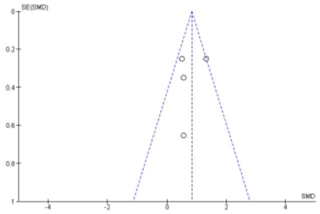

Egger's Intercept = -1.21, p = .70

c. Delayed Recall Test (MCI)

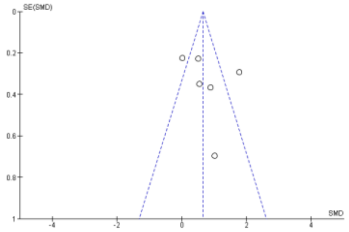

Egger's Intercept = 0.81, p = .46

d. Working Memory (MCI)

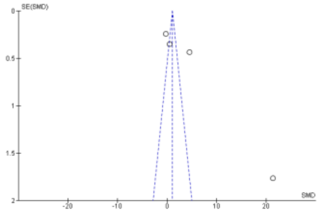

Egger's Intercept = 4.07, p = .06

e. Verbal Fluency (MCI)

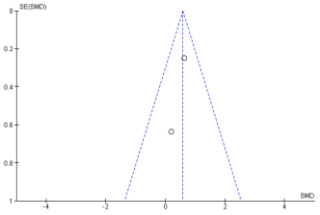

Egger's test was unable to perform due to the number of studies was small.

f. Complex Attention - Trail Making Test-A (MCI)

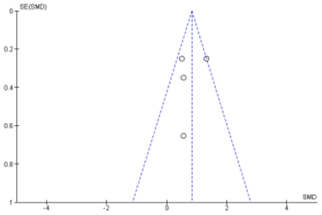

Egger’s Intercept (after Trim-and-fill) = -0.56, p =. 59

g. Executive Function - Trail Making Test-B (MCI)

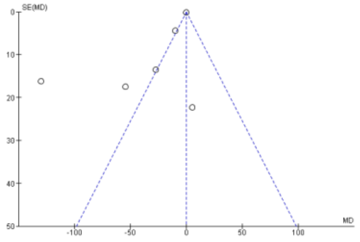

Egger’s Intercept = -1.72, p = .15

h. Global Cognitive Function (Dementia)

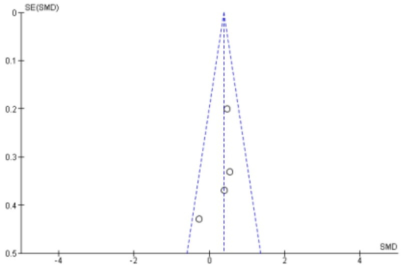

Egger’s Intercept = -1.39, p =.36

Supplementary Figure 2. Effects of Exergaming Intervention on Learning & Memory in People with Mild Cognitive Impairment

2a. Immediate Recall Test

	Exergaming			Control				Std. Mean Difference
**Study or Subgroup**	**Mean**	**SD**	**Total**	**Mean**	**SD**	**Total**	**Weight**	**IV, Random, 95% CI**
Anderson-Hanley 2012	0.77	0.53	38	0.06	0.53	41	32.4%	1.33 [0.84, 1.82]
Liao 2020	4.94	2.85	18	3.13	3.5	16	24.3%	0.56 [-0.13, 1.25]
Mrakic-Sposta 2018	0.68	1.4	5	0.06	0.25	5	10.9%	0.56 [-0.72, 1.84]
Tarnanas 2014	4.9	6.41	32	2.3	3.54	34	32.4%	0.50 [0.01, 0.99]
**Total (95% CI)**			**93**			**96**	**100.0%**	**0.79 [0.31, 1.27]**

Heterogeneity: Tau² = 0.12; Chi² = 6.46, df = 3 (P = 0.09); I² = 54%
Test for overall effect: Z = 3.24 (P = 0.001)

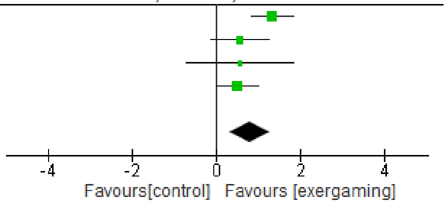

2b. Delayed Recall Test

	Exergaming			Control				Std. Mean Difference
**Study or Subgroup**	**Mean**	**SD**	**Total**	**Mean**	**SD**	**Total**	**Weight**	**IV, Random, 95% CI**
Anderson-Hanley 2012	0.71	1.7	38	0.1	0.28	41	19.6%	0.51 [0.06, 0.95]
Liao 2020	1.45	1.54	18	0.57	1.66	16	16.7%	0.54 [-0.15, 1.22]
Liu 2022	1.2	2.24	16	-0.7	1.95	17	16.3%	0.88 [0.17, 1.60]
Mrakic-Sposta 2018	0.63	0.63	5	0.06	0.35	5	9.5%	1.01 [-0.35, 2.37]
Park 2018	9.31	2.54	39	9.26	4.38	39	19.7%	0.01 [-0.43, 0.46]
Tarnanas 2014	2.8	1.03	32	0.2	1.75	34	18.1%	1.78 [1.20, 2.35]
**Total (95% CI)**			**148**			**152**	**100.0%**	**0.75 [0.20, 1.31]**

Heterogeneity: Tau² = 0.35; Chi² = 23.80, df = 5 (P = 0.0002); I² = 79%
Test for overall effect: Z = 2.67 (P = 0.008)

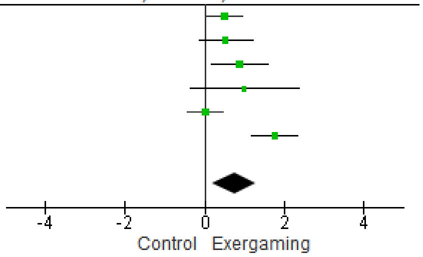

Supplementary Figure 3. Effects of Exergaming Intervention on Working Memory in People with Mild Cognitive Impairment

	Exergaming			Control				Std. Mean Difference
**Study or Subgroup**	**Mean**	**SD**	**Total**	**Mean**	**SD**	**Total**	**Weight**	**IV, Random, 95% CI**
Anderson-Hanley 2012	0.36	0.06	38	-0.83	0.05	41	21.2%	21.41 [17.96, 24.87]
Liu 2022	2.1	2.11	16	0.5	3.21	17	26.3%	0.57 [-0.13, 1.27]
Park 2018	0.4	0.08	39	0.11	0.04	39	26.1%	4.54 [3.68, 5.40]
Tarnanas 2014	0.2	1.12	32	0.4	0.97	34	26.4%	-0.19 [-0.67, 0.29]
**Total (95% CI)**			**125**			**131**	**100.0%**	**5.83 [2.27, 9.39]**

Heterogeneity: Tau² = 12.42; Chi² = 224.47, df = 3 (P < 0.00001); I² = 99%
Test for overall effect: Z = 3.21 (P = 0.001)

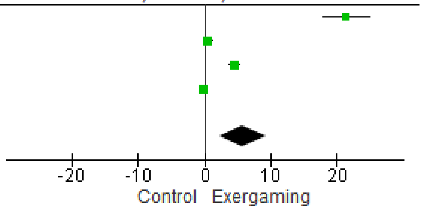

Supplementary Figure 4. Effects of Exergaming Intervention on Verbal Fluency in People with Mild Cognitive Impairment

Study or Subgroup	Exergaming Mean	Exergaming SD	Exergaming Total	Control Mean	Control SD	Control Total	Weight	Std. Mean Difference IV, Random, 95% CI
Mrakic-Sposta 2018	0.28	0.97	5	0.11	0.44	5	13.4%	0.20 [-1.04, 1.45]
Tarnanas 2014	2.8	4.27	32	0.4	3.17	36	86.6%	0.64 [0.15, 1.13]
**Total (95% CI)**			**37**			**41**	**100.0%**	**0.58 [0.12, 1.03]**

Heterogeneity: Tau² = 0.00; Chi² = 0.40, df = 1 (P = 0.53); I² = 0%
Test for overall effect: Z = 2.49 (P = 0.01)

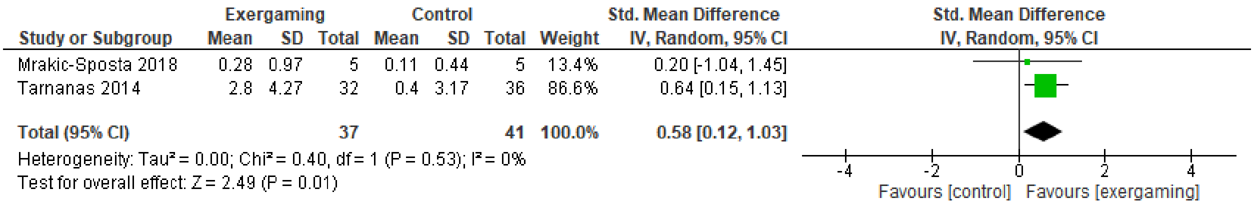

Supplementary Figure 5. Effects of Exergaming Intervention on Complex Attention with Trail Making Test-A in People with Mild Cognitive Impairment

Study or Subgroup	Exergaming Mean	Exergaming SD	Exergaming Total	Control Mean	Control SD	Control Total	Weight	Mean Difference IV, Random, 95% CI
Liao 2019	-9.77	23	21	-7.19	17.85	21	1.2%	-2.58 [-15.03, 9.87]
Liu 2022	-11.2	28.98	18	-1.6	29.18	18	0.5%	-9.60 [-28.60, 9.40]
Mrakic-Sposta 2018	-0.03	0.57	5	0	0.59	5	74.8%	-0.03 [-0.75, 0.69]
Schwenk 2016	-5.8	16.33	11	2.7	14.15	9	1.1%	-8.50 [-21.86, 4.86]
Thapa 2020	-2.1	4.73	34	-0.1	6.05	34	22.3%	-2.00 [-4.58, 0.58]
**Total (95% CI)**			**89**			**87**	**100.0%**	**-0.65 [-2.05, 0.75]**

Heterogeneity: Tau² = 0.55; Chi² = 4.64, df = 4 (P = 0.33); I² = 14%
Test for overall effect: Z = 0.90 (P = 0.37)

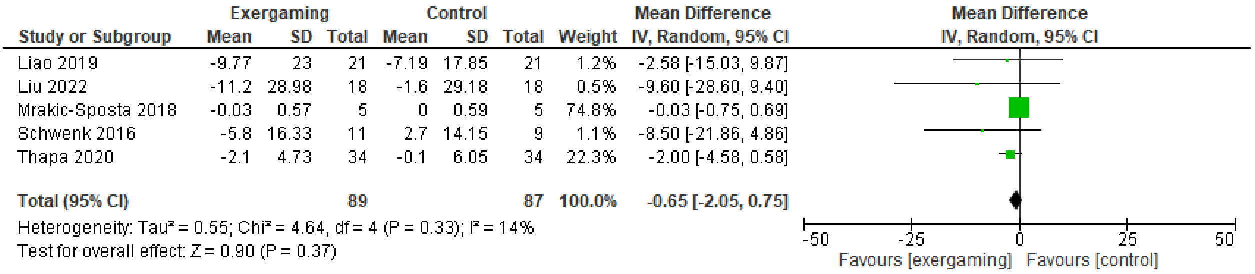

Supplementary Figure 6. Effects of Exergaming Intervention on Executive Function with Trail Making Test-B in People with Mild Cognitive Impairment

Study or Subgroup	Exergaming Mean	Exergaming SD	Exergaming Total	Control Mean	Control SD	Control Total	Weight	Mean Difference IV, Random, 95% CI
Amjad 2019	-2.39	0.2	18	3.78	0.2	20	35.6%	-6.17 [-6.30, -6.04]
Liao 2020	-45.01	37.75	18	-18.13	41.07	16	3.7%	-26.88 [-53.51, -0.25]
Liu 2022	-60.2	48.73	16	-5.7	52.12	17	2.3%	-54.50 [-88.91, -20.09]
Park 2018	-1.31	0.98	39	-1.38	1.21	39	35.5%	0.07 [-0.42, 0.56]
Schwenk 2016	6.4	66.64	11	0.9	28.6	9	1.5%	5.50 [-38.09, 49.09]
Tarnanas 2014	-89.8	78.83	32	40	48.55	34	2.7%	-129.80 [-161.62, -97.98]
Thapa 2020	-5.3	17.15	33	4.7	18.54	33	18.7%	-10.00 [-18.62, -1.38]
**Total (95% CI)**			**167**			**168**	**100.0%**	**-9.71 [-15.13, -4.29]**

Heterogeneity: Tau² = 21.49; Chi² = 656.18, df = 6 (P < 0.00001); I² = 99%
Test for overall effect: Z = 3.51 (P = 0.0004)

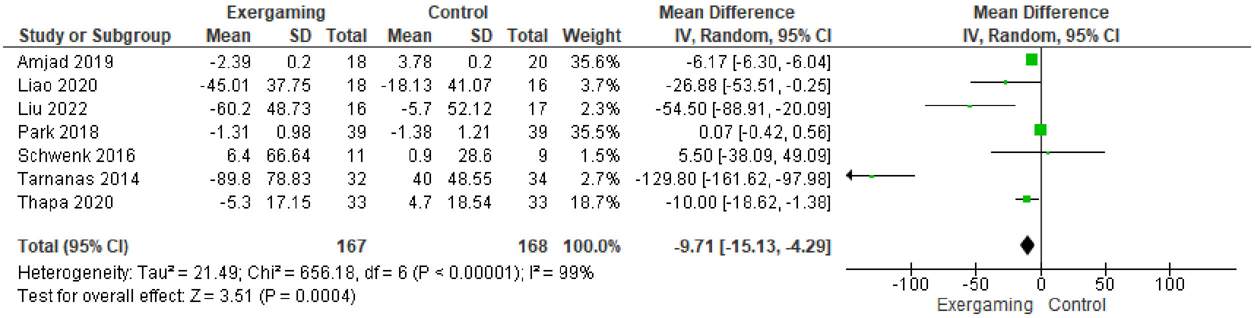

Supplement: Supplementary file 1 — Supplementary table 1,2,3,4, Supplementary Figure 1,2,3,4,5,6 [file 41746_2024_1142_MOESM1_ESM.pdf]
